# Conjunctiva-Associated Lymphoid Tissue (CALT) Reactions to Antiglaucoma Prostaglandins with or without BAK-Preservative in Rabbit Acute Toxicity Study

**DOI:** 10.1371/journal.pone.0033913

**Published:** 2012-03-19

**Authors:** Hong Liang, Christophe Baudouin, Antoine Labbe, Luisa Riancho, Françoise Brignole-Baudouin

**Affiliations:** 1 INSERM, U968, Paris, France; 2 UPMC Univ Paris 06, UMR_S 968, Institut de la Vision, Paris, France; CNRS, UMR_7210, Paris, France; 4 Centre Hospitalier National d'Ophtalmologie des Quinze-Vingts, INSERM-DHOS CIC 503, Paris, France; 5 Assistance Publique - Hôpitaux de Paris Hôpital Ambroise Paré, Service d'Ophtalmologie, Boulogne-Billancourt, France; 6 Université Versailles Saint-Quentin-en-Yvelines, Versailles, France; 7 Université Paris Descartes, Faculté des Sciences Pharmaceutiques et Biologiques, Laboratoire de Toxicologie, Paris, France; The University of Hong Kong, Hong Kong

## Abstract

Conjunctiva-associated lymphoid tissue (CALT) is closely associated with ocular surface immunity. This study investigated the effects of antiglaucoma prostaglandin analogs with or without benzalkonium chloride (BAK) preservative on organized CALT using an acute toxic model. A total of 48 albino rabbits were used and seven groups of treatments were constituted. Solutions (50 µl) of PBS, 0.02%BAK, ^0.02%BAK+^latanoprost, ^0.015%BAK+^travoprost, ^0.005%BAK+^bimatoprost, ^BAK-free^travoprost preserved with the SofZia® system or ^BAK-free^tafluprost were instilled 15 times at 5-min intervals in both eyes. CALT changes were analyzed using *in vivo* confocal microscopy (IVCM), immunohistology in cryosections for detecting MUC-5AC+ mucocytes and CD45+ hematopoietic cells. Antiglaucoma eye drops stimulated inflammatory cell infiltration in the CALT, and seemed to be primarily related to the concentration of their BAK content. The CALT reaction after instillation of BAK-containing eye drops was characterized by inflammatory cell infiltration in the dome and intrafollicular layers and by cell circulation inside the lymph vessels. CD45 was strongly expressed in the CALT after instillation of all BAK-containing solutions at 4 h and decreased at 24 h. The number of MUC-5AC+ mucocytes around the CALT structure decreased dramatically after instillation of BAK-containing solutions. This study showed for the first time the *in vivo* aspect of rabbit CALT after toxic stimuli, confirming the concentration-dependent toxic effects of BAK. IVCM-CALT analysis could be a pertinent tool in the future for understanding the immunotoxicologic challenges in the ocular surface and would provide useful criteria for evaluating newly developed eye drops.

## Introduction

Benzalkonium chloride (BAK), the most widely used preservative in eye drops, has largely been shown to damage the ocular surface of patients treated over the long term. These toxic effects are well documented in the ophthalmological and biomedical literature, and encompass a large variety of mechanisms involving the immune system, conjunctival and corneal epithelia, tear film and most likely corneal nerve sensitivity [1], [2], [3], [4], [5]. In other systems, BAK has already been shown to stimulate and influence local immune regulation. Exposure of mice ears to BAK induced significant B cell activation in the draining lymph nodes (DLN), with an increase in the percentage of B220+ cells [6]. BAK also influences antigen-presenting cells, as BAK was shown in experimental irritant contact dermatitis to induce a state of metabolic activation in a high proportion of epidermal CD1+ Langerhans cells [7].

However, few data are still available concerning BAK and ocular immunology. We have found that long-term use of topical treatments containing BAK stimulated both the Th1 and Th2 systems, based on the overexpression of immune-related chemokine receptors by the conjunctival epithelium [2]. Indeed, ocular surface immunology relies on eye-associated lymphoid tissue (EALT), which includes conjunctiva-associated lymphoid tissue (CALT) and lachrymal drainage-associated lymphoid tissue (LDALT). These systems regulate and concentrate the resident mucosal immune system and are implicated in ocular innate and acquired immunity by detecting antigens and distributing cytokines throughout the ocular surface [8], [9]. CALT is involved in ocular immunology during a large range of physiological and pathological behaviors, such as antimicrobial defense, allergy, allograft rejection or immune tolerance [8]–[10], [11]. In models of ocular pathologies, CALT-like lymphoid aggregates were shown to develop actively during corneal transplantation in rats [12]. In mice, CALT follicles were stimulated in the nictitating membrane, with B/T cells, dendritic cells and macrophages under topical bacterium (*Chlamydia trachomatis*) or bacterium-related protein (cholera toxin) challenges [13].

We have recently shown the *in vivo* aspects of cell reactions in rabbit CALT, after lipopolysaccharide (LPS) or tumor necrosis factor-alpha (TNF) inflammatory stimuli using *in vivo* confocal microscopy (IVCM) and immunohistological assessments [14]. An induction of inflammatory cell infiltration in the dome and in the intrafollicular layers of CALT and an increase of cell circulation inside the lymph node vessels were observed. The present study aimed to explore another type of stimulation on this CALT structure, namely of toxic origin such as that induced by the well-recognized contact irritant BAK, and to compare it with the effects of various BAK-containing antiglaucomatous eyedrops. We mainly focused on the main prostaglandin analogs (PGA) currently available worldwide, as belonging to the major family of antiglaucomatous drugs and as they present different biochemical properties and irritant features [15], [16]. Interestingly, these eyedrops differ from one another in their BAK concentration and may provide interesting comparative information on CALT involvement. Until now the influence of BAK on CALT has not been reported. CALT activation could therefore become an important tool for investigating the immunotoxicological impact of BAK and other preservatives more thoroughly and achieving further refinements in the determination of the toxicological profile of newly developed drugs.

## Methods

### Animals

All experiments were conducted in accordance with the ARVO Statement for the Use of Animals in Ophthalmic and Vision Research. Male albino rabbits aged 2.5 months, weighing 2 kg (New Zealand), were used. A mixture of ketamine (35 mg/kg, Imalgène 500; Merial, Lyon, France) and xylazine (5 mg/kg; Bayer, Puteaux, France) was used to anesthetize the animals by intramuscular injections. We chose rabbit model, because it is the reference animal for standard ocular toxicity testing, and it offers a large area of conjunctiva for observing the CALT structure correctly. Before all experiments, ocular surface integrity was examined using slit-lamp microscopy, and the basal CALT aspect was examined using IVCM. A total of 48 rabbits were used for this toxicological model on antiglaucoma prostaglandin analogs. Two additional normal rabbits were also used for exploring the aspects of the meibomian glands, goblet cells and CALT structures in normal unstimulated rabbits. Four rabbits were used to find the maximal reaction times. The number of rabbits used for each experiment is shown in Table 1.

**Table 1:** Number of rabbits used for each “treatment” condition and for each observation time.

*Treatment*	*dose*	*75 min+2 h+4 h+24 h*	*4 h*	*24 h*
Sterile PBS	15 instillations	2	4	2
0.02%BAK	15 instillations	2	4	2
0.02%BAK+latanoprost	15 instillations		4	2
0.015%BAK+travoprost	15 instillations		4	2
0.005%BAK+bimatoprost	15 instillations		4	2
BAK-free travoprost Z	15 instillations		4	2
BAK-free tafluprost	15 instillations		4	2
No treatment	Normal rabbits	2		

### Normal rabbit whole-mount conjunctiva, impression cytology and cryosections for meibomian gland, goblet cells and CALT structures

In order to better understand the correlations of IVCM images with standard immunohistology, impression cytology (IC) and cryosections were used for exploring normal rabbit meibomian glands, goblet cells and CALT structures.

IC specimens were collected following techniques previously described in normal rabbits in order to observe the goblet cells [17]. Two nitrocellulose membranes (Millipore, Bedford, MA, USA) were applied onto the superior bulbar conjunctiva and then dipped into tubes containing 1.5 ml of cold PBS with 4% paraformaldehyde (PFA) for immunohistology. IC specimens were first incubated with antibodies directed against soluble mucins of the MUC-5AC gene (kindly provided by Jacques Bara, INSERM, University Paris 6, Paris, France) for 1 h at 4°C. Sections were then stained with Alexa Fluor® 488 anti-mouse immunoglobulin (1∶500, Molecular Probes, Eugene, OR, USA). Propidium iodide (PI) was used to stain nuclei red. A confocal microscope (PCM2000; Nikon, Tokyo, Japan) was used to analyze goblet cells on impression cytology.

Two normal rabbits were then sacrificed using an overdose injection of pentobarbital (CEVA Santé Animale, Libourne, France) for preparing the cryosections in order to observe the meibomian glands and CALT structures. The whole-mount conjunctiva with partial eyelids of one of the normal rabbits was dissected and confocal microscopy was used to observe the meibomian glands and conjunctival tissue of an entirely dissected conjunctiva. For the preparation of cryosections, enucleated eyes of the other normal rabbit were fixed in 4% PFA for 4 h at 4°C, and 10 µm cryosections were prepared and incubated with antibodies directed against MUC-5AC products for 1 h at 4°C. Sections were then stained with the secondary antibody and later with PI. Images were digitized with a fluorescence microscope (BX-UCB; Olympus, Melville, NY, USA): particular attention was paid to the distribution of goblet cells around the CALT structure.

### Antiglaucoma eyedrop treatments

Concerning drug testing, eye drops were instilled according to a previously validated acute toxicological model [17], [18]. In order to follow the dynamic activation of CALT using *in vivo* confocal microscopy, four rabbits were anesthetized, two instilled with PBS and two instilled with 0.02%BAK, soon after the last instillation (75 min after the first instillation; first instillation was considered as hour (H) 0) and the CALT changes were observed 75 min, 2 h, 4 h and 24 h after the first instillation.

Seven other groups of three rabbits each (two for 4 h and one for 24 h, based on the first follow-up study findings) were constituted according to the different treatments: 50 µl of sterile PBS, 0.02%BAK or commercial BAK-containing solutions of ^0.02%BAK+^latanoprost (Xalatan®; Pfizer, New York, NY, USA), ^0.0015%BAK+^travoprost (Travatan®, Alcon, Fort Worth, TX, USA), ^0.005%BAK+^bimatoprost (Lumigan®, Allergan, Irvine, CA, USA), and two BAK-free prostaglandin analogs, namely ^BAK-free^travoprost preserved with the SofZia® system (Travatan Z®, Alcon) and ^BAK-free^taflluprost (Taflotan®, Santen, Oy). Using a micropipette, all solutions were applied in both eyes at 5-min intervals a total of 15 times. The CALT structures were observed at 4 h and 24 h. After IVCM analysis, the rabbits were sacrificed for preparing eye cryosections. As previous experiments showed that changes observed after repeated eyedrop instillations were mild in intensity and transient, both eyes received the investigated treatments, in order to reduce the number of animals used and sacrificed. The local ethics committee for animal experimentation of the Faculty of Pharmaceutical and Biological Sciences Paris Descartes University approved the protocol (permit number P2.FBB.072.09).

### CALT *in vivo* confocal microscopy observation

The laser scanning IVCM Heidelberg Retina Tomograph (HRT) II/Rostock Cornea Module (RCM, Heidelberg Engineering GmbH, Heidelberg, Germany) was used for CALT structure investigations [17], [19]. No special dye injection or other special preparation was needed. We observed the lymphoid follicles of the CALT structure according to the previous study [14]: the superior rabbit conjunctiva was slightly turned over; then we moved the IVCM objective delicately over the entire bulbar conjunctiva and partial fornix. The most superficial layer was manually determined as ‘0’ µm, and the depth of CALT was checked manually by the x-y-z position of the optical section. For all eyes, at least three follicles in different areas were recorded and analyzed by IVCM as repeated image captures or as video frames. As previously determined [12], the deepest layer of IVCM visualization was approximately 60–80 µm. To standardize the IVCM analysis of CALT, the layer from 0 to 15 µm was defined as the ‘dome layers’ of CALT and the layers from 15 to 30 µm as the ‘intrafollicular layers’. For video recording, the ‘x–y’ axis (z: 0 µm) and the ‘x–z’ axes of the CALT follicles were recorded separately. The infiltrating inflammatory cells were counted using the Cell Count® program. The final counts of inflammatory cells (lymphocytes, polymorphonuclear cells and dendritiform cells) were the averages of the three follicles of four rabbit eyes.

### CALT immunohistology in cryosections and positive cell counts

At 4 h, the rabbits were euthanized with an overdose injection of pentobarbital. After enucleation, eyes were fixed in 4% PFA for 4 h at 4°C and 10-µm cryosections were prepared and incubated with antibodies directed against rabbit CD45 (1∶50; CBL1412, Cymbus Biotechnology, Chandlers Ford, UK) for 2 h at 4°C. Sections were then incubated with the secondary antibody (Alexa Fluor®488 anti-mouse immunoglobulin, 1∶500) for 1 h. PI was used to stain the nuclei. Images were digitized using an Olympus BX-UCB fluorescent microscope (Olympus, Melville, NY, USA). Immunopositive cells were counted on cryosections from three rabbits per treatment using a 100×100-µm reticle. The center parts of the CALT follicles were counted and three sections were used for each treatment. The results were presented as means and standard deviations for each treatment.

### Statistical analysis

Results were expressed as means ± standard error (SE). The groups for analysis were compared using factorial analysis of variance (ANOVA) followed by the Fisher method (Statview V; SAS Institute Inc., Cary, NC, USA).

## Results

### IVCM observation of Meibomian glands, Goblet cells and CALT structure

**Figure 1:**
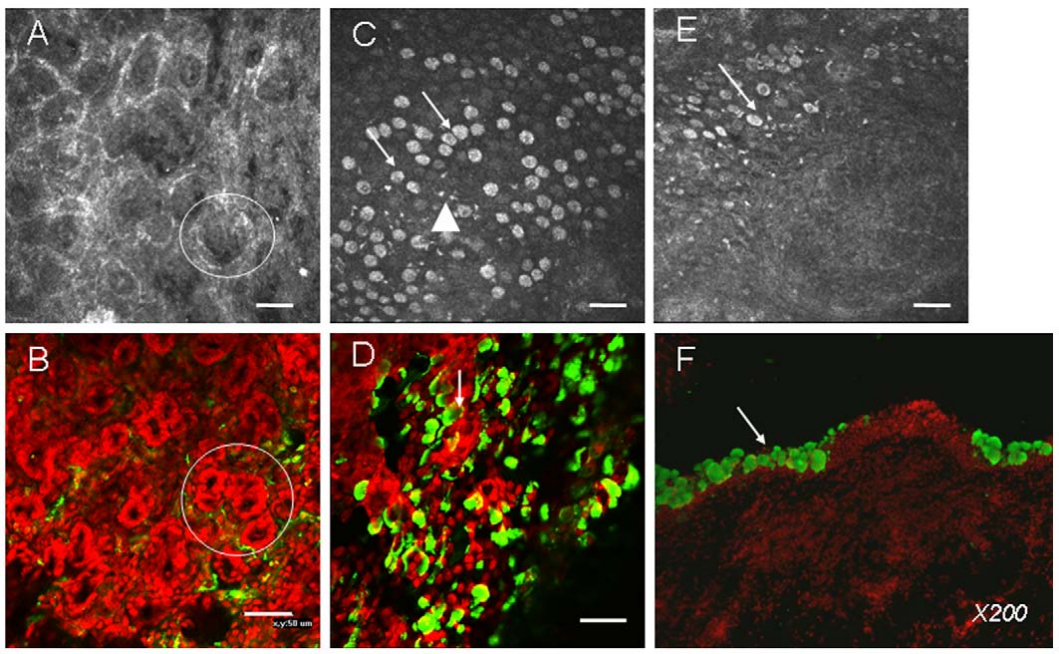
The morphological correlation between IVCM analysis (A, C, E) and immunohistology (B, whole-mount conjunctiva; D, impression cytology; F, cryosections) for observing the microstructure of superior conjunctiva in normal rabbits. Bar: 50 µm. IVCM observation for meibomian acinar glands (Fig. A, circle) is similar to that of whole-mount conjunctiva with partial eyelids (Fig. B, circle). Goblet cells presented large hyperreflective, round or oval aspects (arrows in C for IVCM and D for impression cytology). Several white hyperreflective inflammatory cells were found (triangles). The CALT structure under the IVCM presented a round/oval aspect (Fig. E) with the goblet cells surrounding them. Immunohistology of MUC-5AC in cryosections confirmed this distribution of goblet cells (F).

In IVCM, the aspect of rabbit meibomian glands (Fig. 1A) was similar to that found in the whole-mount conjunctiva (Fig. 1B). They appeared morphologically as acinar glands, mainly located at the tarsal conjunctiva, with a darker central hole in each gland corresponding to the orifice of the gland duct.

The IVCM goblet cell or mucocyte analysis appeared difficult due to the various aspects found in individual rabbits. In some normal rabbits, we could clearly visualize the large hyperreflective, round/oval cells most probably representing soluble mucin-secreting goblet cells (Fig. 1C, arrows). They always accumulated together as mass with some white hyperreflective inflammatory cells among them (rectangles). Using immunohistology in impression cytology, these cells were characterized as MUC-5AC-positive goblet cells (Fig. 1D, arrows) disseminated among the conjunctival epithelium whose nuclei are stained with PI in red.

The CALT structure presented a round/oval aspect, which is quite different from other conjunctiva structures (Fig. 1E, arrow). As Knop et al. mentioned in previous studies, the goblet cells were located around the follicles and were never found in the dome layers. MUC-5AC immunohistology in rabbit conjunctiva transversal cryosections confirmed this distribution of goblet cells surrounding the follicles (Fig. 1F, arrow).

### Dynamic observation of CALT activation 75 min, 2 h and 4 h after instillations of 0.02% BAK

**Figure 2:**
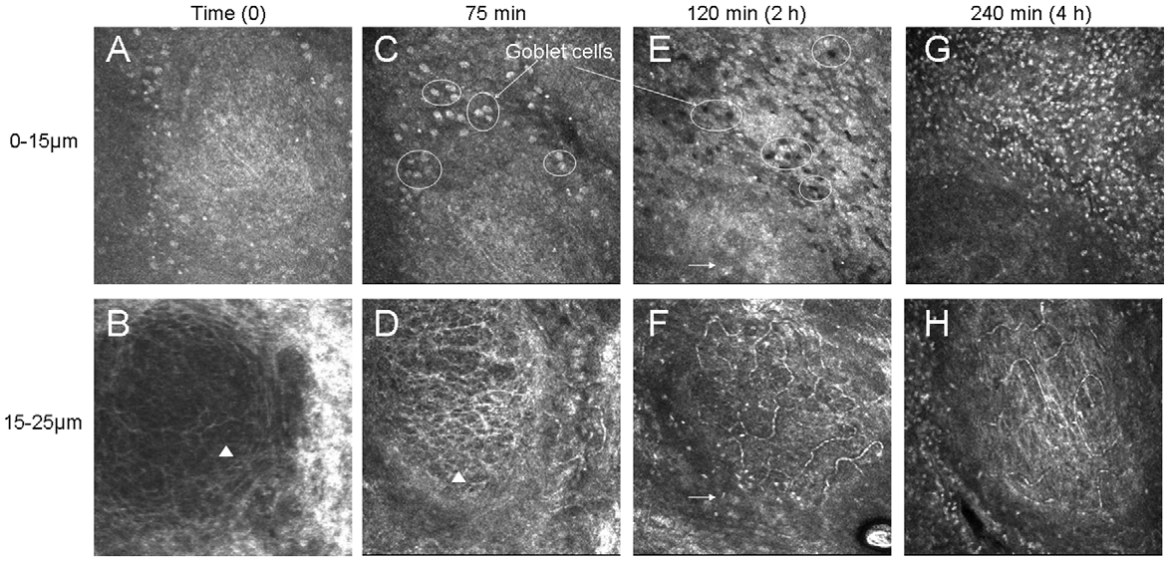
IVCM analysis of rabbit CALT, 75 min (A, B), 2 h (C, D) and 4 h (E, F) after the first instillation of ^0.02%BAK+^latanoprost in dome layers (A, C, E: 0–15 µm) and in intrafollicular layers (B, D, F: 15–30 µm). All images: 400 µm×400 µm. Note the disappearance of goblet cells around the CALT structure with the increase of inflammatory cell infiltration in dome layers and the activation of lymphatic vessels with the increase of inflammatory cell infiltration in the intrafollicular layers.

Compared to the untreated rabbit at time zero (Fig. 2A and 2B), after 15 instillations (75 min from the first instillation) of 0.02%BAK, the CALT structure was not yet activated (Fig. 2C and 2D). At that time, many goblet cells were found around the CALT follicles (Fig. 2C, circles), which presented white hyperreflective aspects. In the deeper layers of follicles, no special lymphatic vessel activation was found (Fig. 2D).

Two hours after the first instillation, the CALT activated significantly after the treatment of 0.02%BAK (Fig. 2E and 2F). At the dome layers, some hyperreflective inflammatory patterns were observed (Fig. 2E, arrow). At the same time, the goblet cells, always found around the CALT, lost their white hyperreflective aspect and darkened, which was quite different from their aspect at 75 min (Fig. 2C). In the intrafollicular layers, the activation of lymphatic vessels was observed (Fig. 2F). The infiltration of inflammatory cells was noted, not only inside the intrafollicular structure, but also in the parafollicle area (arrows). In the intrafollicular layers, by video mode, we observed the circulation of white patterns, possibly inflammatory/immune cells, inside the lymphatic vessels at a high speed. Figure 2F showed one image of the video. The infiltrated inflammatory cells were quite different from standard hyperreflective gray patterns (assumed to be resting lymphocytes) initially contained in normal CALT (Fig. 2B and 2D, rectangle), as they presented larger and brighter aspects. Since IVCM functions with the contrast between the structures, the original gray patterns were not easy to distinguish at this time due to the strong contrast of infiltrated inflammatory cells, and we could not determine whether such cells were still in the CALT follicles, had migrated or had morphologically changed after activation.

Four hours after the first instillation, the activation of CALT and the infiltration of inflammatory cells were more obvious, reaching maximal levels (Fig. 2G). At the dome of the superficial layer (Fig. 1G), numerous inflammatory cells were observed outside (1138.2±78.5 cells/mm^2^) and also inside the follicle (56.8±6.9 cells/mm^2^). Hyperreflective and hyporeflective aspects of goblet cells were no longer observed at this time. Numerous inflammatory cells could also be noted in the intrafollicular layers (Fig. 2H). They circulated inside lymphatic vessels, which were clearly recorded, and more inflammatory cells were found in the parafollicle areas. This CALT activation decreased at 24 h.

The same dynamics were also observed following the PBS instillations: no special changes were found in the dome and in the intrafollicular layers at 75 min, 2 h and 4 h with the persistence of normal hyperreflective goblet cells (data not shown).

### Rabbit CALT activation after antiglaucoma eye drop instillations

**Figure 3:**
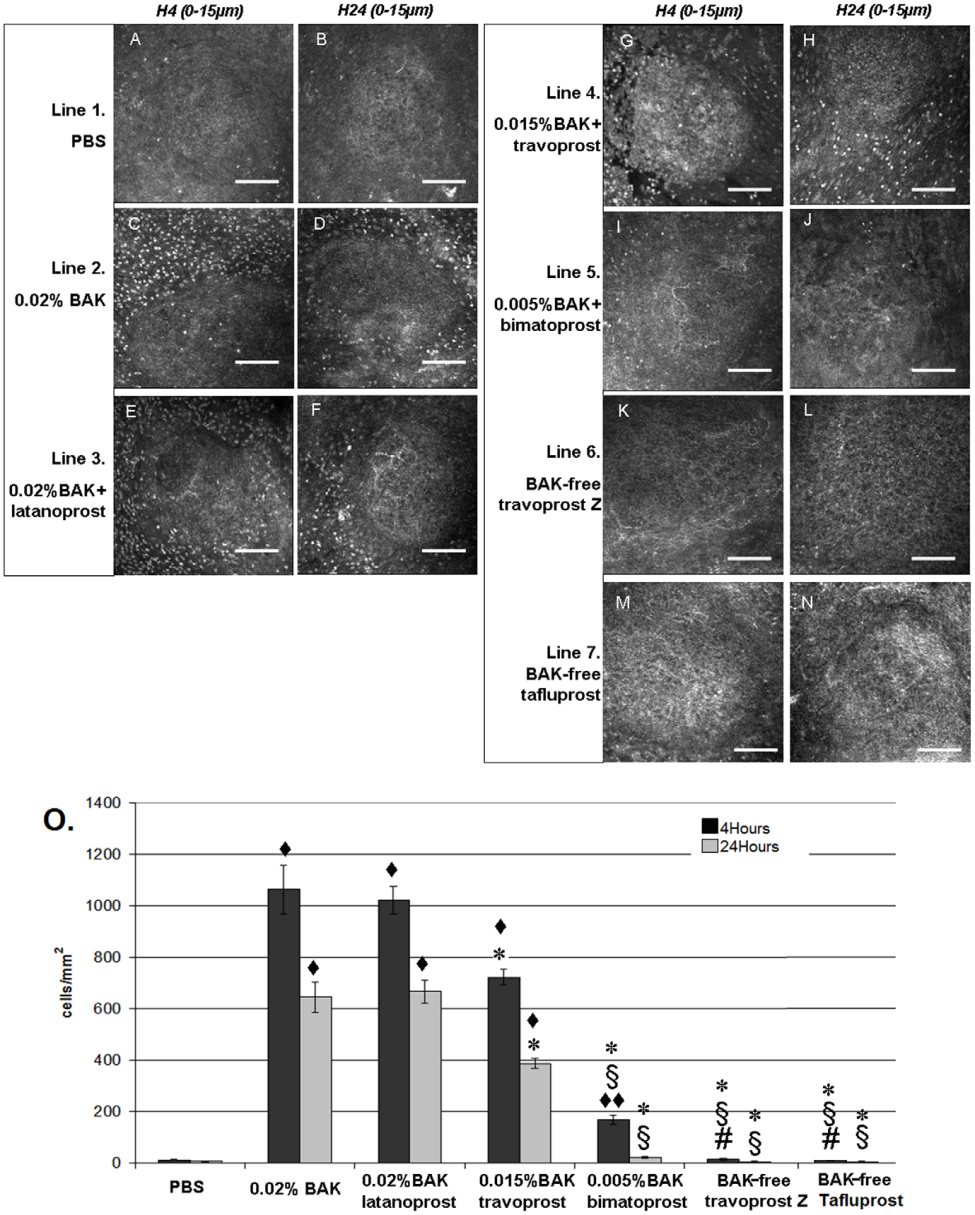
IVCM analysis in rabbit CALT dome layers (0–15 µm) after 15 instillations of PBS (A, B), 0.02%BAK (C, D), ^0.02%BAK+^latanoprost (E, F), ^0.015%BAK+^travoprost (G, H), ^0.005%BAK+^bimatoprost (I–J), ^BAK-free^travoprost (K, L) or ^BAK-free^tafluprost (M, N) at 4 h (A, C, E, G, I, K, M) and at 24 h (B, D, F, H, J, L, N). Note the hyperreflective patterns of inflammatory cells, especially after the application of 0.02BAK%, ^0.02%BAK+^latanoprost and ^0.015%BAK+^travoprost (IVCM images: bar, 100 µm). The inflammatory cells were counted using Cell Count software attached to the IVCM (O). ♦ *P*<0.0001 compared to PBS and ♦♦ *P*<0.03 compared to PBS; * *P*<0.0001 compared to ^0.02%BAK+^latanoprost and 0.02%BAK groups; § *P*<0.0001 compared to ^0.015%BAK+^travoprost group; # *P*<0.03 compared to ^0.005%BAK+^bimatoprost group.

Figure 3 presents the IVCM images of the dome layers of CALT follicles after 15 instillations with the different eye drops at 4 h (Fig. 3A, C, E, G, I, K, M) and 24 h (Fig. 3. B, D, F, H, J, L, N). At 4 h, PBS instillations did not induce any obvious changes in CALT structure (Fig. 3A with 12.8±3.3 cells/mm^2^, *P*<0.0001 compared to all other groups, Fig. 3O for cell count), while 0.02%BAK (Fig. 3C with 1063.2±94.9 cells/mm^2^) and ^0.02%BAK+^latanoprost solution (Fig. 3E with 1021.2±52.7 cells/mm^2^) (*P*<0.0001 compared to all other groups for 0.02%BAK and ^0.02%BAK+^latanoprost groups) induced substantial inflammatory cell infiltration. IVCM made it possible to register *in vivo* the x–y axis and x–z axis aspects of CALT activation, as shown in video S1 and video S2 after the 15 instillations of ^0.02%BAK+^latanoprost. Application of ^0.015%BAK+^travoprost also induced numerous hyperreflective inflammatory cells around CALT follicles at 4 h (Fig. 3G; 724.5±30.3 cells/mm^2^) (*P*<0.0001 compared to all other groups). ^0.005%BAK+^Bimatoprost induced less inflammatory cell infiltration at 4 h (Fig. 3I; 169.3±20.0 cells/mm^2^, x–y axis in video S3 and x–z axis in video S4) (*P*<0.001 compared to PBS and ^BAK-free^travoprost and ^BAK-free^tafluprost groups at H4). When ^BAK-free^travoprost or ^BAK-free^tafluprost was instilled onto the rabbit ocular surface, no particular changes in CALT were found when compared with PBS treatment (less than 20 cells/mm^2^). Twenty-four hours after eye drop applications, the inflammatory cell infiltration levels decreased in all groups (Fig. 3. B, D, F, H, J, L and Fig. 3O for cell counts). However, the inflammatory infiltrations persisted especially after the instillations of 0.02%BAK, ^0.02%BAK+^latanoprost, ^0.015%BAK+^travoprost at moderate levels with 648.5±58.45 cells/mm^2^, 667.67±44.47 cells/mm^2^, 388.33±18.15 cells/mm^2^ respectively. At this time, there was no difference between the PBS, ^0.005%BAK+^bimatoprost, ^BAK-free^travoprost and ^BAK-free^tafluprost groups, with fewer than 25 cells/mm^2^.

### Immunohistology analyses of CD45-positive cells in cryosections

**Figure 4:**
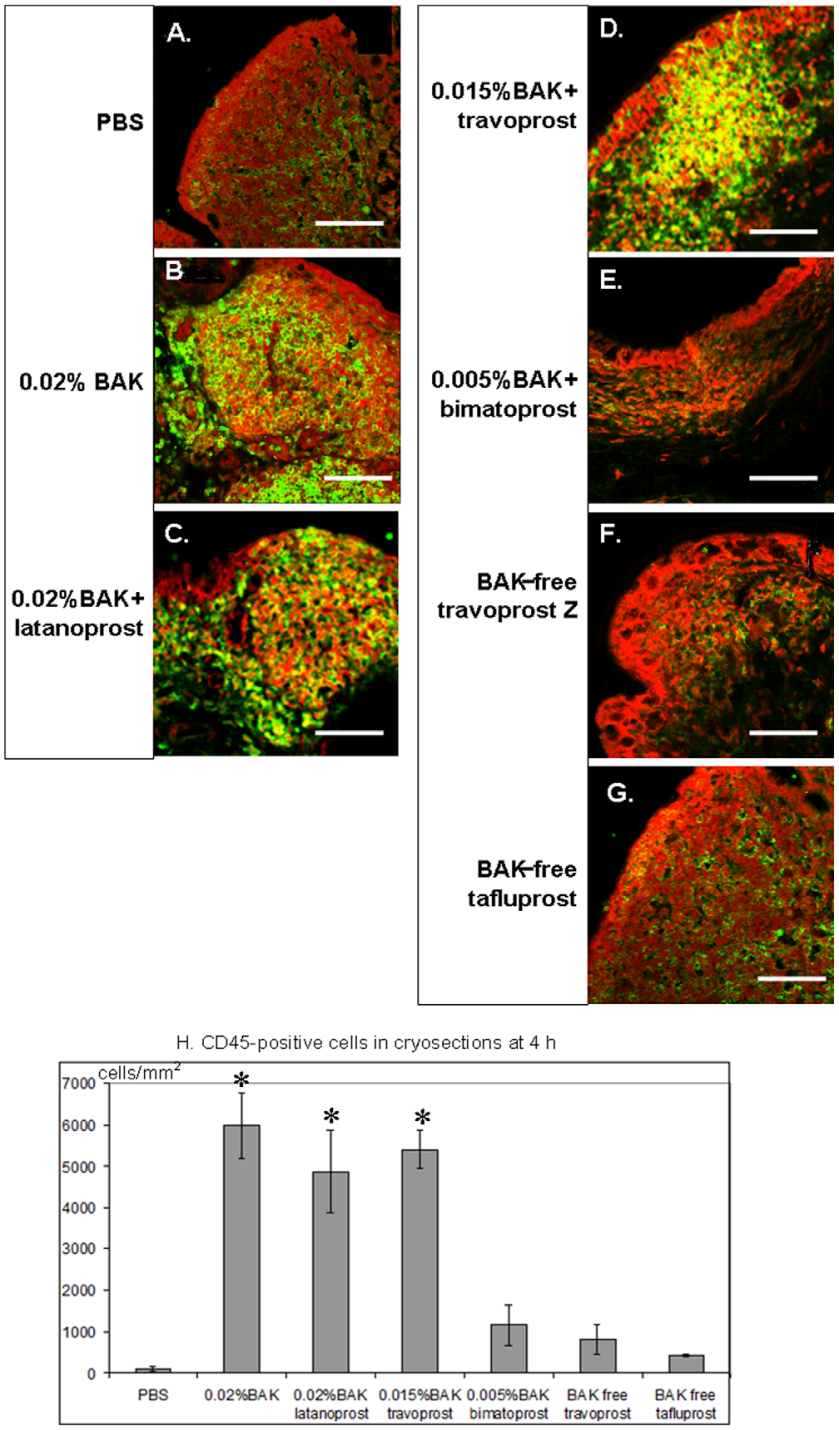
Immunofluorescence staining of CD45 in the cryosections at 4 h: very few CD45+ cells were observed after PBS applications (A), the strongest expression of CD45+ cells in CALT follicles was found after instillation of 0.02%BAK (B), ^0.02%BAK+^latanoprost (C) and ^0.015%BAK+^travoprost (D). Scattered CD45+ cells were also found after the instillation of ^0.005%BAK+^bimatoprost (E), ^BAK-free^travoprost Z (F) or ^BAK-free^tafluprost (G). (Bar, 100 µm). CD45+ cell counts in cryosections are presented in Figure 4H. * *P*<0.0001 compared to PBS, *P*<0.001 compared to ^0.005%BAK+^bimatoprost, and *P*<0.0002 compared to ^BAK-free^travoprost Z or ^BAK-free^tafluprost.

At 4 h, a faint expression of CD45 was observed in the cryosections (Fig. 4) after the instillation of sterile PBS (100±58 cells/mm^2^, Fig. 4A). In the same time, we observed the strongest expression of CD45+ lymphocyte-resembling cells after the instillations of 0.02%BAK (5967±800 cells/mm^2^, Fig. 4B), ^0.02%BAK+^latanoprost (4867±993 cells/mm^2^, Fig. 4C) and ^0.015%BAK+^travoprost (5400±458 cells/mm^2^, Fig. 4D) (*P*<0.0001 compared to PBS, *P*<0.001 compared to ^0.005%BAK+^bimatoprost, and *P*<0.0002 compared to ^BAK-free^travoprost Z and ^BAK-free^tafluprost for the three groups, Fig. 4H). These CD45+ cells accumulated in the dome and intrafollicular layers of the CALT follicles and were also scattered in the conjunctival stroma adjacent to CALT follicles. ^0.005%BAK+^bimatoprost (Fig. 4E), ^BAK-free^travoprost (Fig. 4F) and ^BAK-free^tafluprost (Fig. 4G) caused only faint infiltration of CD45+ cells (1167±491 cells/mm^2^ and 800±361 cells/mm^2^, respectively, with no significant differences with PBS, Fig. 4H).

### Immunohistological analyses of MUC-5AC+ mucocytes

**Figure 5:**
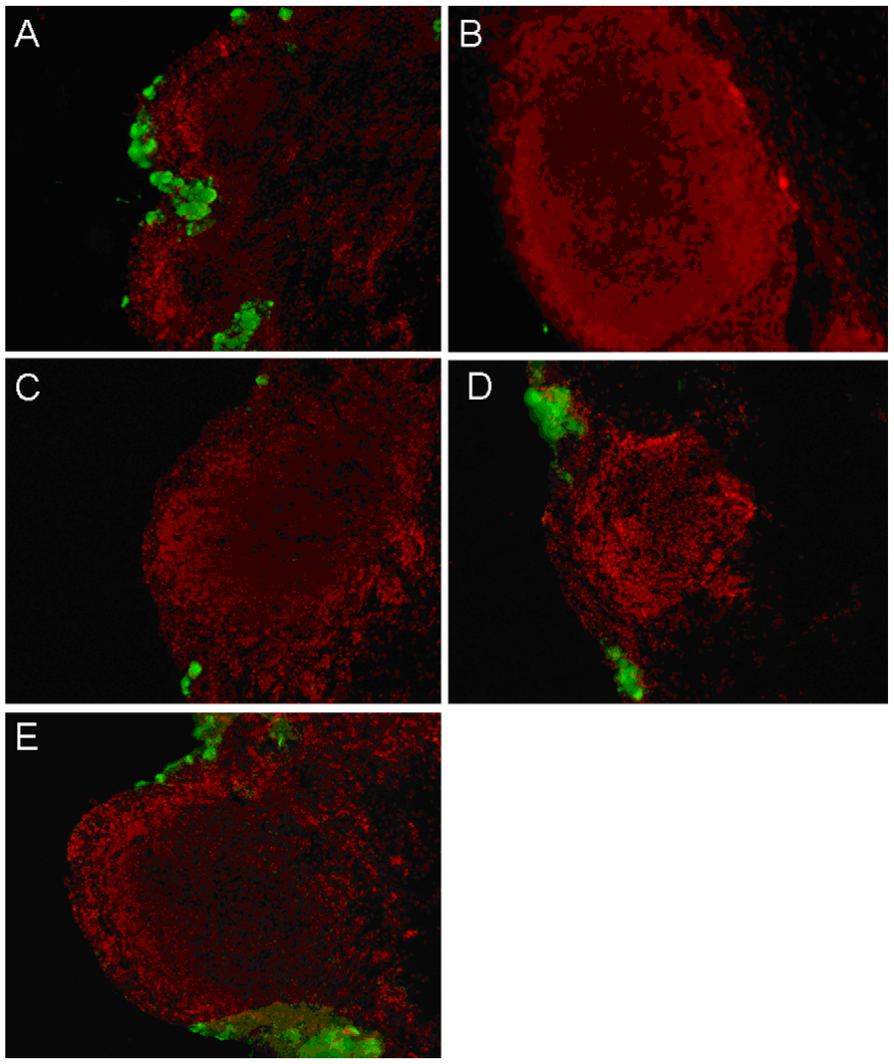
Immunofluorescence staining of MUC-5AC in the cryosections at 4 h: normal goblet cell patterns after PBS applications (A). The follicle size increased, and no or rare goblet cells were observed after the instillations of 0.02%BAK (B) or ^0.02%BAK+^latanoprost (C) with unstructured green paths. BAK-free travoprost Z (D) and BAK-free tafluprost (E) presented almost normal MUC-5AC staining. (Images ×200).

After the instillation of PBS, MUC-5AC+ mucocytes persisted, as in normal rabbits, located around the follicles (Fig. 5A), even between two neighboring CALTs. No goblet cells were observed in the dome layers. After the instillations of all BAK-containing solutions, mucocytes around the CALT dramatically decreased with unstructured green patterns (Fig. 5B and 5C). However, after the instillation of either BAK-free solution, MUC-5AC-positive cells were normally found around the CALT follicles (Fig. 5D for ^BAK-free^travoprost and Fig. 5E for ^BAK-free^tafluprost), even though they were larger than the control ones.

## Discussion

This study investigated the *in vivo* aspects of CALT after topical antiglaucoma treatments with prostaglandin analog containing different concentrations of BAK. BAK is a quaternary ammonium commonly used in eye drops where it prevents microbial contamination and is also widely found in the environment. It is a well-recognized irritant that also has sensitizing properties and could even be, albeit rarely, a contact allergen [20]. Many studies have reported its toxic effects to ocular surface epithelia, causing ocular surface epithelia disruption and apoptosis, stimulating inflammatory reactions, impairing corneal nerves, destroying goblet cells and even enhancing fibrosis in the subconjunctival spaces [5]. Considering these adverse effects most particularly observed in long-term topical treatments such as in glaucoma therapy, BAK-free formulations were developed to be safer for the ocular surface. Recently, we have conducted toxicological studies on two new BAK-free formulations of prostaglandins, one containing a SofZia ionic buffer named travoprost Z and the other, called tafluprost, which is fully preservative-free. BAK-free travoprost was shown to improve cell viability and reduced apoptosis compared with BAK-containing latanoprost or travoprost [21], and preservative-free tafluprost has confirmed its good tolerance both *in vitro* and *in vivo* [22]–[23]. These current experimental results were in accordance with previous *in vitro* and *in vivo* studies, proving that the damage caused to ocular surface tissues, especially to cornea and conjunctiva epithelia, was primarily related to the BAK concentration [24]–[26].

IVCM has now been widely used by researchers and ophthalmologists to explore the ocular microstructures. Nowadays, after having thoroughly explored the corneal tissue in normal and pathological settings, we succeeded in analyzing conjunctival epithelium, goblet cells, meibomian glands as well as CALT. The development of the IVCM made it possible to observe this latter distinctive immunological structure with no specimen injection or preparation. We investigated an active CALT *in vivo* using IVCM by characterizing the infiltration of inflammatory cells, the absence or pathological aspects of the surrounding goblet cells and the activation of lymphatic vessels in the intrafollicular structures. IVCM was thus not only found to be suitable for morphological observation, but could also be used for the analysis of the sole part of the immune system that is directly accessible to observation. IVCM analysis has been used in glaucomatous patients treated with chronic BAK-containing eye drops by showing ocular surface alterations with the reduction of the density of superficial epithelial cells, the activation of stromal keratocytes, and the reduction in sub-basal nerves and increase in nerve tortuosity [4]. Confirmed by an *ex vivo* study using flat-mount immunofluorescence, Peebo et al. identified the corneal lymph vessels in a rat neovascularization model using the same *in vivo* confocal microscopy: the morphology of lymph vessels was highly specific and enabled subsequent monitoring of lymphatic activity, including the dynamics of leukocyte movement within lymphatics [27].

Several ocular problems such as dry eye, uveitis and scleritis are closely associated with systemic autoimmune disorders. Contributing significantly to the study of corneal nerves, IVCM has been proposed as a noninvasive method for accurately diagnosing and assessing the progression of diabetic neuropathy [28]. Research exploring the conjunctiva-associated immune system has great potential to contribute to a better understanding of ocular surface immunity, systemic problems as well as new vaccine strategies [20], [29]. This *in vivo* study has confirmed the presence of CALT in rabbit eyes and its activation following toxic stimuli using a noninvasive technique. We followed this dynamic *in vivo* analysis by observing the same animal throughout all the experiments, before and after the instillations of ophthalmological solutions, thus avoiding variations between animals. In addition, the information provided by IVCM, including the topography, size and inflammatory cell infiltration, goblet cell counts inside and outside the CALT structures, accurately reflects the activation status of the whole ocular surface and could become important criteria for evaluating the cytotoxicity of newly developed drug formulations. IVCM analyses have already been used for testing new formulations, such as cationic emulsions [30]. They were useful for toxicological purposes and showed that CALT may be stimulated after toxic challenge, consistent with the widely demonstrated proinflammatory effects of antiglaucoma drugs and their preservatives [1], [3]. In our toxicological model, the inflammatory cell infiltration in CALT seemed to be primarily related to the BAK concentration. These immunoinflammatory changes in CALT, and possibly EALT and LDALT, may actively participate in the strong inflammatory and apoptotic reactions observed after applications of these BAK-containing eye drops [31].

In humans, we highlighted the presence of CALT in patients suffering from severe allergic reactions in a previous study [14]. This structure could certainly constitute a new ocular surface entity that could be included in the IVCM-ocular surface scoring system for future immunological and toxicological experiments as well as for the exploration of ocular surface disorders in human pathologies.

## Supporting Information

Video S1 X–Y axis in IVCM analysis of CALT structure 4 h after instillations of ^0.02%BAK+^latanoprost. The depth of IVCM is maintained at 0 µm. A live three-dimensional visualization of a rabbit CALT follicle *in vivo* shows the inflammatory cell infiltration, especially around but also inside the follicles.
(AVI)

Video S2 X–Z axis in IVCM analysis of CALT structure 4 h after instillations of ^0.02%BAK+^latanoprost. The depth of IVCM analysis decreases from 0 µm to 30 µm. A live visualization of a rabbit CALT follicle shows the appearance of lymphatic vessels.
(AVI)

Video S3 X–Y axis in IVCM analysis of CALT structure 4 h after instillations of ^0.005%BAK+^bimatoprost. The depth of IVCM is maintained at 0 µm. The rabbit CALT follicle shows less inflammatory cell infiltration when compared to that of ^0.02%BAK+^latanoprost.
(AVI)

Video S4 X–Z axis in IVCM analysis of CALT structure 4 h after instillations of ^0.015%BAK+^bimatoprost. The depth of IVCM decreases from 0 µm to 30 µm. The rabbit CALT follicle shows less activation when compared to that of ^0.02%BAK+^latanoprost.
(AVI)
